# O-linked N-acetylglucosamine transferase promotes cervical cancer tumorigenesis through human papillomaviruses E6 and E7 oncogenes

**DOI:** 10.18632/oncotarget.10112

**Published:** 2016-06-16

**Authors:** Minjun Kim, Yoon Sook Kim, Hwajin Kim, Min Young Kang, Jeongsook Park, Dong Hoon Lee, Gu Seob Roh, Hyun Joon Kim, Sang Soo Kang, Gyeong Jae Cho, Ji Kwon Park, Jin Won Cho, Jeong Kyu Shin, Wan Sung Choi

**Affiliations:** ^1^ Department of Anatomy and Convergence Medical Science, Institute of Health Sciences, Gyeongsang National University School of Medicine, Jinju, Gyeongnam, Republic of Korea; ^2^ Department of Obstetrics and Gynecology, Gyeongsang National University School of Medicine, Jinju, Gyeongnam, Republic of Korea; ^3^ Department of Integrated OMICS for Biomedical Science, Graduate School, Yonsei University, Seoul, Republic of Korea

**Keywords:** O-GlcNAcylation, host cell factor 1, OGT, E6 and E7

## Abstract

O-linked N-acetylglucosamine (O-GlcNAc) transferase (OGT) increases O-GlcNAc modification (O-GlcNAcylation), and transcriptional co-regulator host cell factor 1 (HCF-1) is one of OGT targets. High-risk Human Papillomaviruses (HPVs) encode E6 and E7 oncoproteins, which promote cervical cancer. Here, we tested whether O-GlcNAc modification of HCF-1 affects HPV E6 and E7 expressions and tumorigenesis of cervical cancer. We found that depleting OGT with OGT-specific shRNA significantly decreased levels of E6 and E7 oncoproteins, and cervical cancer tumorigenesis, while OGT overexpression greatly increased levels of E6 and E7 oncoproteins. Notably, OGT overexpression caused dose-dependent increases in the transcriptional activity of E6 and E7, and this activity was decreased when HCF-1 was depleted with HCF-1-specific siRNA. Moreover, OGT depletion reduced proliferation, invasion, and metastasis in cervical cancer cells. Further, high glucose enhanced the interaction between OGT and HCF-1, paralleling increased levels of E6 and E7 in cervical cancer cells. Most importantly, we found that reducing OGT in HeLa cells caused decreased tumor growth in vivo. These findings identify OGT as a novel cellular factor involved in E6 and E7 expressions and cervical cancer tumorigenesis, suggesting that targeting OGT in cervical cancer may have potential therapeutic benefit.

## INTRODUCTION

Cervical cancer is one of the leading cause of cancer-related deaths worldwide among young women and caused by a persistent infection with human papillomaviruses (HPVs) [1], and current treatment options are insufficient.

O-linked N-acetylglucosaminylation (O-GlcNAcyl ation) is a reversible posttranslational modification of serine/threonine residues [2–4]. O-linked N-acetyl-glucosamine (O-GlcNAc) transferase (OGT) mediates O-GlcNAcylation of various proteins implicated in various disorders, and couples nutrient excess, an important risk factor, to protein activities and cellular function. Notably, increased O-GlcNAcylation is a hallmark in various cancers [5–7]. The increased glucose flux through hexosamine biosynthetic pathway (HBP) results in accumulation of UDP-O-linked N-acetyl-glucosamine (UDP-O-GlcNAc), which is the donor sugar nucleotide used by OGT in the process of O-GlcNAcylation. Importantly, OGT is required for tumor growth and colony formation in vitro [8], and invasion and metastasis in vivo [9, 10].

HPVs are human pathogens that infect cutaneous or mucosal epithelia causing warts [11, 12]. High-risk HPV E6/E7 expression is required for the induction and maintenance of the transformed phenotype, and rate limiting for cervical cancer development [13]. Because of frequent integration of the viral genome into host cell chromosomes, E6 and E7 are consistently expressed in HPV-associated cancers, playing a critical role in cervical cancer. However, the mechanisms underlying the regulation of E6 and E7 expression in host cells remain elusive.

Host cell factor 1 (HCF-1) was originally isolated as a transcription co-factor involved in oncogenic viral reactivation processes at the latent stage, and later found to play a critical role in tumor formation [14]. HCF-1 undergoes a unique mode of limited proteolysis and the proteolytic processing of HCF-1 is required to coordinate HCF-1 function during cell cycle progression [14]. Recently, the crosstalk between O-GlcNAcylation and the proteolytic cleavage of HCF-1 was identified; O-GlcNAcylation is required for proper maturation of HCF-1 and subsequent HCF-1-mediated regulation of cell cycle, while HCF-1 is stabilized by O-GlcNAcylation [15–17]. Although the effects of GlcNAcylation on some tumor-associated proteins are known, the roles of GlcNAcylation in cervical cancer remain largely unknown. In this study, we investigated whether O-GlcNAcylation regulates HPV E6 and E7 expressions and cervical cancer progression. We found that O-GlcNAc modification of HCF-1 enhances transcriptional activity of E6 and E7, and OGT is required for cervical cancer progression. Additionally, we found that high glucose increased O-GlcNAc modification of HCF-1 and E6/E7 levels in cervical cancer cells.

## RESULTS

### The levels of O-GlcNAcylation, OGT, E6, E7 and O-GlcNAcylated HCF-1 are elevated in cervical cancer

**Figure 1:**
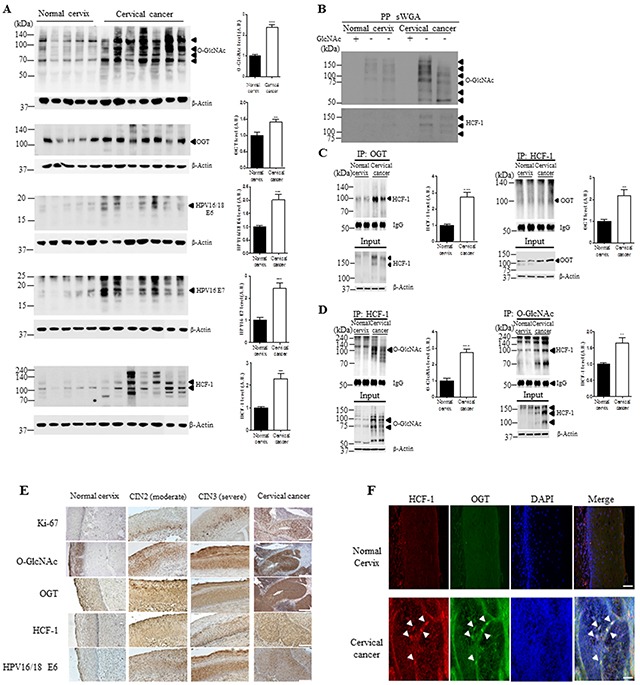
The levels of O-GlcNAcylation, OGT, E6, E7 and O-GlcNAcylation of HCF-1 levels are elevated in cervical cancer tissues **A.** Representative Western blot and quantification of O-GlcNAc, OGT, E6, E7 and HCF-1 in normal cervical (n=5) or cervical cancer (n=7) tissues. Band intensity was normalized to β-actin. Data are presented as mean ± SEM. **P<0.05, ***P<0.001 by *t* test. **B.** Cell lysates were precipitated using agarose beads coupled to sWGA (PP sWGA) and the precipitates were immunoblotted with an anti-O-GlcNAc – or -HCF-1 antibody. For control, the inhibitory monosaccharide GlcNAc was added during sWGA-lectin-affinity purification. Data are representative of at least 3 independent experiments. Binding of OGT **C.** or O-GlcNAc **D.** to HCF-1. Representative immunoblots and quantification of co-immunoprecipitated HCF-1 to OGT or O-GlcNAc in normal cervical or cervical cancer tissues. Tissue lysates were subjected to immuno-precipitation (IP) with an anti-OGT- or - O-GlcNAc antibody and immunoblotted with an anti-HCF-1 antibody. Densitometry of co-immunoprecipitated HCF-1 to OGT or O-GlcNAc was normalized to IgG. Data are presented as mean ± SEM. (n=3 cervical tissues per group). **P < 0.005, ***P < 0.0001 by *t* test. **E.** Representative cervical tissue sections stained with an antibody against Ki-67, O-GlcNAc, OGT, HCF-1, E6 or E7 in the normal cervical, CIN2/3 (moderate/severe) and cervical cancer tissues. **F.** Representative images of double immunofluorescence staining for OGT and HCF-1 plus 4′,6-diamidino-2-phenylindole (DAPI) for nuclear localization. Scale bar, 200 μm.

Because the OGT and O-GlcNAcylation levels are elevated in various cancers, and OGT partners with transcriptional regulator such as HCF-1, and proteolytic processing of HCF-1 is required for activating the function of HCF-1 [15, 16, 18], we examined the O-GlcNAcylation and HCF-1 levels in cervical cancer tissues by western blot and immunohistochemistry analysis. Baseline characteristics of the patients and histology results of the tissues are summarized in Supplementary Table S1. We found that levels of O-GlcNAc and OGT were significantly elevated in HPV16/18-positive E6- and E7-expressing cervical cancer tissues compared to normal cervical tissues (Figure 1A, *P* < 0.0001 and *P* < 0.005, respectively). Further, since cervical carcinogenic mechanism mainly depends on the expression of E6 and E7 oncoproteins, which neutralize cellular tumor suppressor function [19], we measured levels of E6 and E7 through western blot analysis. As expected, we found that E6 and E7 protein levels were significantly enhanced in cervical cancer tissues compared to normal cervical tissues (Figure 1A, *P* < 0.001 and *P* < 0.0005, respectively). Moreover, HCF-1 was significantly increased in cervical cancer tissues compared to normal cervical tissues (Figure 1A, *P* < 0.0001), although the cleavage patterns are slightly different among the patients. As well, in order to determine that the antibody truly detected the sugar modification, succinylated wheat germ agglutinin (sWGA) affinity purification was run. For control, the inhibitory monosaccharide GlcNAc was added during sWGA-lectin-affinity purification to show all true carbohydrate modified proteins disappear. Indeed, we found that O-GlcNAc antibody truly detected the sugar modification because O-GlcNAc mostly disappeared with the inhibitory monosaccharide GlcNAc added during sWGA-lectin-affinity purification (Figure 1B). As well, O-GlcNAcylated HCF-1 precipitated using sWGA disappeared with GlcNAc added during sWGA-lectin-affinity purification (Figure 1B). Furthermore, we examined the interaction between OGT and HCF-1 to determine whether HCF-1 is O-GlcNAcylated in cervical cancer. Immunoprecipitation assays showed that the interaction between OGT and HCF-1 was greatly increased in cervical cancer tissues compared to normal cervical tissues (Figure 1C, *P* <0.0005 or *P*<0.005). Consistently, HCF-1 showed greater O-GlcNAc modification in cervical cancer tissues compared to normal cervical tissues (Figure 1D, *P*<0.0001 or *P*<0.005). These results suggest that the increased interaction between OGT and HCF-1 may cause an increase in O-GlcNAcylated HCF-1 in cervical cancer tissues compared to normal cervical tissues. Consistently, immunohistochemical analysis showed that levels of O-GlcNAc, OGT, HCF-1, and HPV16/18 E6 were significantly increased, particularly in the intermediate layer of cervical cancer tissues compared to normal cervical tissues, paralleling staining of Ki-67 (Figure 1E), a DNA proliferation marker increased in cervical intra-epithelial neoplasia (CIN) [20]. Double immunofluorescence staining of OGT and HCF-1 revealed co-localization of OGT and HCF-1 in the nuclear region of cervical cancer tissues (Figure 1F). These results suggest that O-GlcNAcylation of HCF-1 is associated with increased DNA proliferation in cervical cancer tissues, and OGT may target specific downstream pathways to elicit pathogenic responses in cervical cancer formation.

### O-GlcNAc, OGT, E6 and E7 levels are upregulated in HPV16/18-positive cervical cancer cell lines

**Figure 2:**
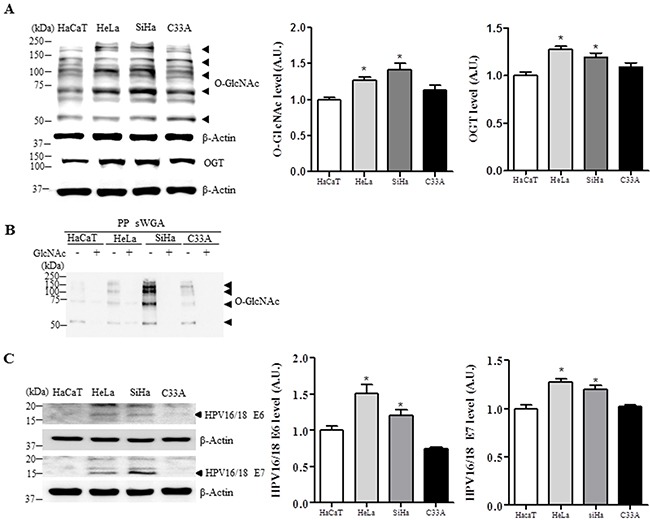
Levels of O-GlcNAc, OGT, E6 and E7 are elevated in HPV-type 16/18-positive human cervical cancer cell lines Representative Western blot and quantification of O-GlcNAc, OGT **A.** E6 and E7 **C.** in control (HaCaT) or cervical cancer cell lines (HeLa, SiHa, and C33A). Band intensity was normalized to β-actin. Data are presented as mean ± SEM. *P<0.05 by *t* test. **B.** Cell lysates were precipitated using agarose beads coupled to sWGA (PP sWGA) and the precipitates were immunoblotted with an anti-O-GlcNAc antibody. For control, the inhibitory monosaccharide GlcNAc was added during sWGA-lectin-affinity purification. Data are representative of at least 3 independent experiments.

We further examined O-GlcNAc levels in several human cervical cancer cell lines or HaCaT cells as a keratinocyte control. Global O-GlcNAc and OGT levels were increased in HeLa and SiHa HPV-positive cervical cancer cell lines compared to C33A and HaCaT HPV-negative cell lines (Figure 2A, *P* <0.05). Moreover, sWGA affinity purification showed that the antibody truly detected the sugar modification, because with the inhibitory monosaccharide GlcNAc added during sWGA-lectin-affinity purification, O-GlcNAc mostly disappeared (Figure 2B). Further, E6 and E7 protein levels were significantly increased in HeLa and SiHa cervical cancer cells compared to HaCaT control cells (Figure 2C, *P* < 0.05).

### Glucose causes an increase in levels of OGT, O-GlcNAc, HCF-1, and E6/E7 in cervical cancer cells

**Figure 3:**
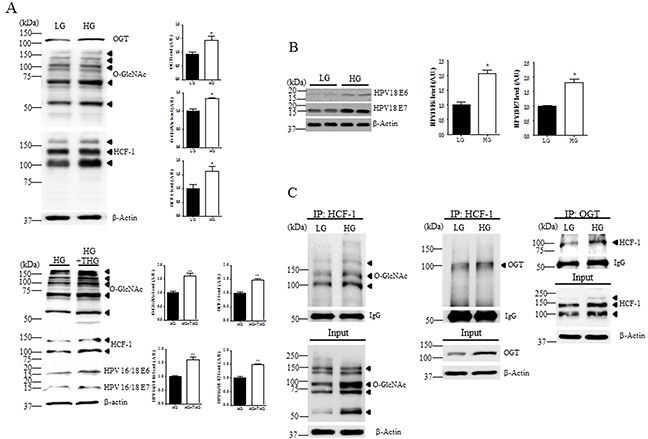
Levels of O-GlcNAc, OGT, HCF-1, E6 and E7 are increased in cervical cancer cells exposed to high glucose Representative Western blot and quantification of O-GlcNAc, OGT and HCF-1 **A.** and E6 and E7 **B.** in HeLa cervical cancer cells treated or not with OGA inhibitor thiamet-G (THG). HeLa cells were incubated in low (LG, 5 mM) or high glucose medium (HG, 25 mM). Band intensity was normalized to β-actin. Data are presented as mean ± SEM. *P<0.05, **P < 0.005 by *t* test. **C.** Representative immunoblots and quantification of co-immunoprecipitated O-GlcNAc or OGT to HCF-1 in HeLa cells exposed LG or HG. The cell extracts were subjected to immunoprecipitation (IP) with an anti-HCF-1 antibody, followed by immunoblot analysis with an anti-O-GlcNAc or -OGT antibody.

Based on the hypothesis that the magnitude of O-GlcNAc modification of intracellular proteins correlates with extracellular glucose levels [21, 22], and hypeprglycemia may be an important cancer risk factor, we examined HeLa cells exposed to low or high glucose. Western blot analysis of HeLa cells in high glucose showed significant increase in O-GlcNAc, OGT, HCF-1, E6 and E7 compared to low glucose (Figure 3A and 3B, *P* < 0.05). To further demonstrate the role of elevated O-GlcNAcylation, we checked the effect of OGA inhibitor thiamet–G on HCF-1 and E6/E7expression levels. As expected, HCF-1 and E6/E7expression levels in HeLa cells exposed to high glucose with thiamet-G were significantly increased compared to those without thiamet–G (Figure 3A, *P* < 0.005). Furthermore, to investigate whether the altered interaction of O-GlcNAc or OGT with HCF-1 in cervical cancer tissues is attributable to glucose availability, we examined the interaction of HCF-1 with O-GlcNAc or OGT in cervical cancer cells exposed to low or high glucose by immunoprecipitation assay. Notably, we found that the interaction between HCF-1 and OGT, and levels of O-GlcNAcyled HCF-1 were greatly increased in HeLa cells exposed to high glucose compared to low glucose (Figure 3C), suggesting that HCF-1 O-GlcNAcylation due to high glucose may lead to altered levels of E6/E7 in cervical cancer cells.

### OGT regulates HCF-1 O-GlcNAcylation, and E6 and E7 expression in cervical cancer cells

**Figure 4:**
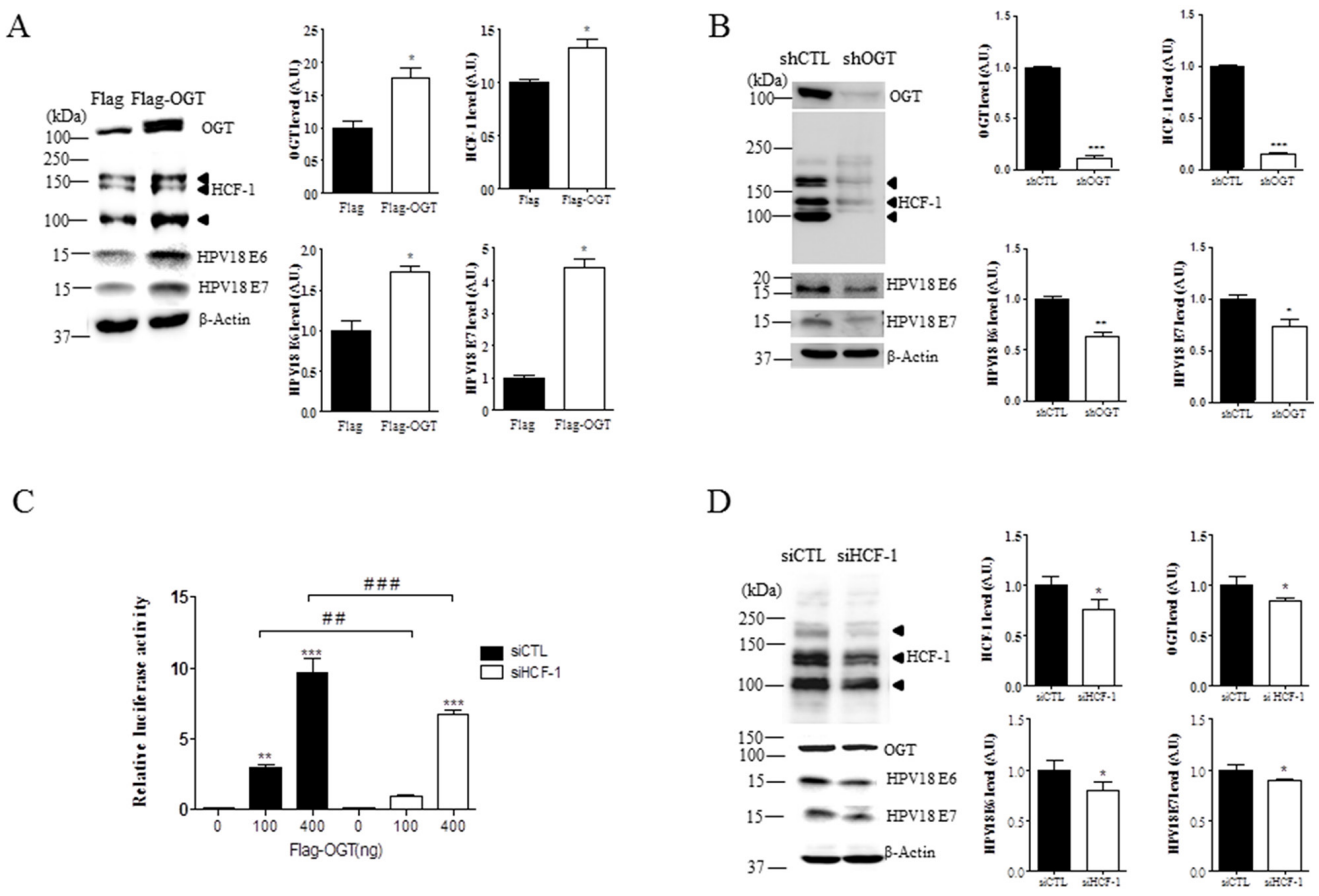
OGT regulates O-GlcNAcylation of HCF-1, and transcriptional activity of E6 and E7 in cervical cancer cells **A.** Representative Western blot and quantification of HCF-1, E6, and E7 in HeLa cells with Flag or Flag-OGT. Band intensity was normalized to β-actin. Data are presented as mean ± SEM. *P < 0.05 by *t* test. **B.** Representative Western blot and quantification of O-GlcNAc, HCF-1, E6 and E7 in HeLa cells treated with non-targeting (shCTL) or OGT-specific shRNAs (shOGT). Band intensity was normalized to β-actin. Data are presented as mean ± SEM. *P < 0.05, **P < 0.005, ***P < 0.0001 by *t* test. **C.** HeLa cells were co-transfected with E6/E7 reporter constructs in combination with Flag or Flag-OGT expression plasmids, and 24 h later the cells were treated with non-targeting or HCF-1-specific siRNA (siCTL or siHCF-1), and then analyzed for luciferase activity. Data are presented as mean ± SEM. **P<0.05, ***P<0.005, ^##^*P* <0.005 ^###^*P* <0.0001 by ANOVA. **D.** Representative Western blot and quantification of OGT, E6 and E7 in HeLa cells treated with non-targeting or HCF-1-specific siRNA (siCTL or siHCF-1). Band intensity was normalized to β-actin. Data are presented as mean ± SEM. *P < 0.05 by *t* test.

Because HCF-1 is stabilized by O-GlcNAcylation [16], and a transcription co-factor involved in oncogenic viral reactivation processes, playing a critical role in tumor formation, we tested the effects of OGT overexpression on HCF-1 in HeLa cervical cancer cells transfected with Flag or Flag-OGT. We found that HCF-1 levels were significantly increased when OGT is overexpressed, paralleling increased levels of E6 and E7 (Figure 4A, *P* < 0.05), suggesting that increased OGT promotes E6 and E7 induction. Moreover, depleting OGT levels with OGT-specific shRNA significantly reduced levels of HCF-1 in HeLa cervical cancer cells as shown by western blot analysis (Figure 4B, *P* < 0.0001), supporting a role for OGT in stabilizing HCF-1 protein. Most importantly, OGT deletion significantly decreased E6 or E7 expression levels in HeLa cells (Figure 4B, *P* < 0.005 or *P* < 0.05, respectively), suggesting that OGT may induce HPV E6 or E7 expression through HCF-1 O-GlcNAcylation. To support these results, we tested whether the transcriptional activity of the E6/E7 promoter is dependent on OGT and HCF-1. The transcriptional activity of the E6/E7 promoter was measured through an HPV18-LCR-luciferase reporter system. By co-expressing a HPV18 luciferase reporter (HPV18-Luc) along with Flag or Flag-OGT, we found that OGT was able to induce HPV18-Luc activity (Figure 4C). Notably, we found that OGT overexpression caused a dose-dependent (100 and 400 ng) increase in the transcriptional activity of the E6/E7 promoter in HeLa cells (Figure 4C, *P* < 0.05). Furthermore, to examine whether HCF-1 affects the OGT-dependent transcriptional activity of the E6/E7 promoter we treated HeLa cells transfected with flag or flag-OGT with scrambled or HCF-1-specific siRNA. Importantly, we found that the luciferase activity was significantly decreased when HCF-1 was depleted with HCF-1-specific siRNA (siHCF-1) (Figure 4C, *P* < 0.005). Moreover, we found that HeLa cells with siHCF-1 exhibited a significant reduction in the levels of OGT, E6 and E7, compared to the cells treated with non-targeting siRNA (Figure 4D, *P* < 0.05). Together, these results suggest that OGT is required for the HCF-1-mediated E6/E7 transcriptional activation in cervical cancer cells.

### OGT depletion inhibits cell proliferation, colony formation, and invasion in cervical cancer cells and reduces tumor growth *in vivo*

**Figure 5:**
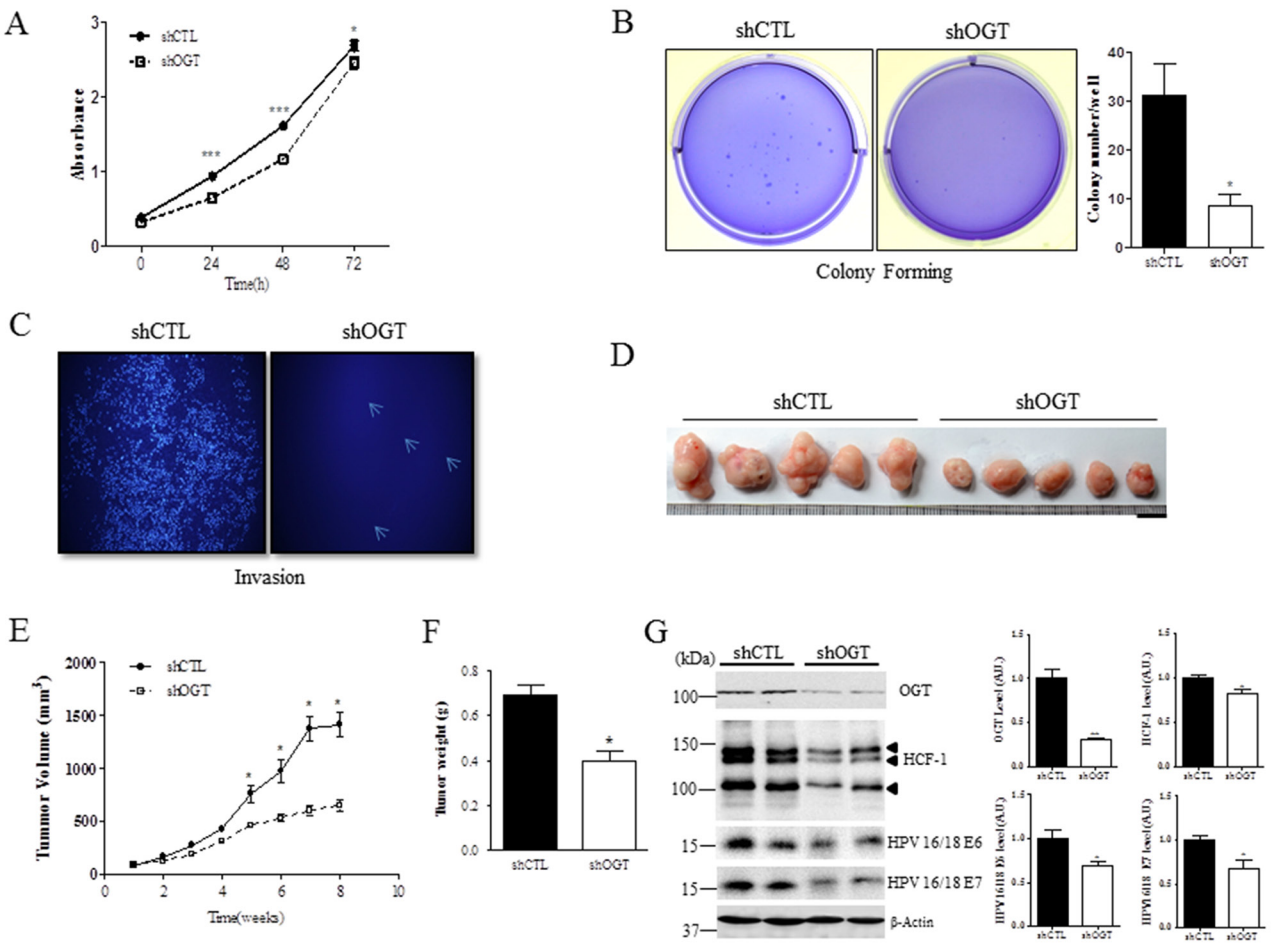
OGT depletion decreases cell proliferation, colony formation, and invasion, and reduces tumor formation *in vivo* **A.** Cell proliferation at the indicated times after non-targeting (shCTL) or OGT-specific shRNA (shOGT) treatment was measured with MTT assay in HeLa cells. Data are presented as mean ± SEM. *P < 0.05, ***P < 0.0001 by *t* test. **B.** HeLa cells were infected with control (shCTL) or OGT shRNA (shOGT). Cell colonies were stained with crystal violet. Data are presented as mean ± SEM. *P < 0.05 by *t* test. **C.** Invasion ability of HeLa cells after non-targeting (shCTL) or OGT-specific shRNA (shOGT) treatment was measured on transwell inserts. **D.** Representative tumors in mice 8 weeks after injection. Scale bar, 1 mm. **E.** Mean tumor volume (mm^3^) of nude mice injected with HeLa cells with the indicated treatment is shown at the indicated week. Data are presented as mean ± SEM. *P < 0.05 by *t* test. **F.** Mean tumor weight of nude mice injected with HeLa cells with the indicated treatment is shown when mice were killed. Data are presented as mean ± SEM. *P < 0.05 by *t* test. **G.** Representative Western blot and quantification of HCF-1, E6 and E7 in tumors of nude mice after injection of HeLa cells treated with non-targeting (shCTL) or OGT-specific shRNA (shOGT). Band intensity was normalized to β-actin. Data are presented as mean ± SEM. *P < 0.05, **P < 0.01 by *t* test.

Because depleting OGT with shRNA affects E6 and E7 protein expression in cervical cancer cells (Figure 4B), and cervical carcinogenic mechanism mainly depends on the expression of E6 and E7, we hypothesized that OGT depletion may affect cell proliferation and metastasis in cervical cancer. To test this we measured cell viability, colony formation, and invasion in HeLa cervical cancer cells. Notably, OGT depletion significantly inhibited cell viability, as measured by the MTT assay (Figure 5A, *P* < 0.05 or *P* < 0.0001), and markedly reduced the ability of cells to grow in three-dimensional culture compared to the control cells (Figure 5B, *P* < 0.05). Moreover, OGT depletion greatly suppressed cell invasion compared to the control cells (Figure 5C). Further, to test whether OGT is required for tumor growth in vivo, HeLa cells stably expressing either control or OGT shRNA were injected subcutaneously into the right flank of nude (Nu/Nu) mice. Tumor growth was visualized over time utilizing caliper. Reducing OGT in HeLa cells caused decreased tumor growth in vivo (Figure 5D). Cells in the context of OGT depletion decreased tumor volume (Figure 5E, *P* < 0.05) and tumor weight (Figure 5F, *P* < 0.05) compared to cells with non-targeting shRNA. Thus, these results showed that OGT regulates tumor growth in vivo, suggesting that OGT may contribute to cervical cancer malignancy by promoting tumor growth. Additionally, to show the relationship between OGT, HCF-1 and E6/7, we performed western blot analysis on HCF-1 and E6/E7 in tumors of nude mice after injection of HeLa cells treated with non-targeting (shCTL) or OGT-specific shRNAs (shOGT). As expected, we found that levels of HCF-1 and E6/E7 were significantly decreased in tumor cells of nude mice injected with HeLa cells with shOGT compared to those with shCTL (Figure 5G, *P* < 0.05 or *P* < 0.01).

## DISCUSSION

Several tumor-associated proteins are GlcNAcylated proteins [23]. In this study, we showed that O-GlcNAc modification of HCF-1 increases HPV E6/E7 oncogenes in cervical cancer, showing the relationship between HCF-1-mediated increase in HPV E6/E7 oncogenes and nutrient flux. Previous studies have reported on upregulation of OGT and elevated O-GlcNAc modification in several cancers [8, 10, 24], but not in cervical cancer. Here, we showed that OGT activates HPV *E6/E7* transcription though O-GlcNAc modification of HCF-1. Cervical cancer arises in persistent HPV lesions through the action of E6 and E7, via reactivation of HPV infection [25–27]. Because E6 and E7 are involved in epigenetic reprogramming, a critical step in tumorigenesis and metastasis [27, 28], regulation of E6 and E7 expression would be a promising strategy for treating HPV-induced cervical cancer. Although previous studies have shown that targeting HPV E6/E7 mRNAs with siRNA could effectively knock down their expression and induce apoptotic cell death in HPV-positive cell lines [28, 29], siRNAs only block HPV E6/E7 mRNAs temporally, and they do not attack the HPV DNA in the nuclei, which is a store of escape mutants that cause resistance to siRNA application. Accordingly, instead of targeting RNA, designing upstream transcription factor to regulate E6 and E7 oncoproteins could be a potential strategy for preventing cervical cancer tumorigenesis.

Notably, we showed here the regulatory mechanism underlying E6 and E7 expression in cervical cancer. Because repeated and enhanced expression of E6 and E7 results in cell immortalization and transformation [30, 31], it is intriguing that O-GlcNAc modification of HCF-1 increases E6 and E7 transcription in cervical cancer, where E6 and E7 can be targeted for immune therapy.

O-GlcNAc modification of HCF-1 by OGT is a critical step in proteolytic cleavage of HCF-1 [15, 32]. The heterodimer formed by the cleaved HCF-1 fragments enhances histone remodeling and the transcription of cancer-inducing genes. A previous study suggests that OGT/HCF-1 complex is a glucose sensor because glucose availability modulates gluconeogenesis through OGT/HCF-1 complex, and that O-GlcNAc signaling serves as a nexus between nutrient flux or insulin resistance and diabetes [33]. As such, we found that O-GlcNAc modification of HCF-1 in cervical cancer cells were elevated under high glucose conditions. Therefore, our findings suggest that the virus-mediated E6/E7 oncogenic induction and cervical cancer tumorigenesis may be enhanced in patients with nutrient excess, providing insights into the relationship between glucose availability and cervical cancer.

Recent studies reported that deletion of OGT blocks the growth and proliferation of several types of cancer cells [10, 34, 35]. Consistently, we found that OGT deletion attenuates the growth and proliferation of cervical cancer cells. Moreover, OGT deletion suppressed invasion, metastasis, and colony formation, suggesting that O-GlcNAc modification may not only regulate transcriptional processes relevant to cervical cancer, but also may affect the trafficking of cell adhesion molecules that are important to metastasis. Most importantly, in nude mice, tumor volume from HeLa cells treated with OGT–specific shRNA was significantly reduced compared to those with non-targeting shRNA. Therefore, these effects mediated by O-GlcNAc modification suggest that OGT could be a therapeutic target for cervical cancer. OGT may be selectively inhibited by OSMI-1 which reduced viral yields over 1,000-fold at the concentration of 50μM [36, 37] or a commercially available OGT inhibitor ST045849 [38].

Overall, our finding that O-GlcNAc modification of HCF-1 increases E6 and E7 in cervical cancer cells and in vivo strongly suggests an important role for the nutrient sensor OGT in human cervical cancer.

## MATERIALS AND METHODS

### Patient tumor samples

Human cervical tissue samples were collected from patients undergoing cervical biopsies and loop electrosurgical excision procedures, with patient characteristics summarized in Supplementary Table S1. This study was approved by the Human Research Protection Office at the Gyeongsang National University. Forty cervical tissue samples were selected from the tissue archives of the Department of Pathology at Gyeongsang National University Hospitals. These tissues had been designated as being/containing atypical squamous cells of undetermined significance, low-grade squamous intraepithelial lesion, or high-grade intraepithelial lesion based on data from liquid-based cytological smear analyses. The patients were examined by colposcopy and HPV sampling and then, biopsy specimens were obtained. The patients were high-risk HPV16/18-positive and aged between 29 and 84 years (mean, 57.1 years). Histological results were defined as no cervical intra-epithelial neoplasia (CIN 0), mild dysplasia (CIN 1), or moderate to severe dysplasia (CIN 2/3), based on a consensus review by two experienced pathologists. The final cohort of study participants was classified into three groups: (i) HR-HPV (−) normal cervix; (ii) HR-HPV (+) CIN 2/3; (iii) HR-HPV (+) cancer. Tissue samples from normal subjects, CIN2 (moderate), CIN3 (severe), or cervical cancer patients were embedded in paraffin blocks and sectioned for staining, or used for protein extraction. Informed consent was obtained from all participants, and the study was approved by the Ethics Committee of our hospital (IRB No. 2014-10-024-001).

### Cell lines

Cervical cancer cell lines HeLa (HPV-18-positive), SiHa (HPV-16-positive), C-33A (HPV-negative), and a human keratinocyte cell line (HaCaT) were obtained from American Type Culture Collection (Manassas, VA, USA). HeLa and HaCaT cells were maintained in Dulbecco's modified Eagle's medium (DMEM), and SiHa and C33A cells were maintained in Minimum Essential Media (MEM) in 5% CO_2_ at 37°C. All media were supplemented with 10% fetal bovine serum (FBS; Invitrogen, Carlsbad, CA, USA), 100 μg/mL streptomycin, and 100 units/mL penicillin (Invitrogen). Cells were grown in low (5 mM) or high (25 mM) glucose media.

### Plasmid transfection and luciferase reporter assay

Cells were grown to ~90% confluence in a 24-well plate and transfected with the plasmids pCMV-Tag (FLAG control) or pCMV-Tag OGT using Lipofectamine 2000 (Invitrogen, Carlsbad, CA, USA) according to the manufacturer's instructions. For luciferase reporter assay, HeLa cells were co-transfected with HPV18-LCR-Luc or pGL3-basic (Promega, Madison, WI, USA). HeLa cells were transfected with Flag-OGT at the indicated amounts (0, 100, or 400 ng) along with 300 ng of β-gal and 400 ng of pGL3-basic or HPV18-LCR-Luciferse reporter plasmid in HeLa cells. After 24 h, cells were washed with cold phosphate-buffered saline (PBS) and lysed in the lysis buffer provided in the Luciferase Assay System (Promega). Transfection efficiency was normalized using the β-Galactosidase Enzyme Assay System (Promega), and luminescence was measured using an Infinite M200 Pro microplate reader (Tecan, Maennedorf, Switzerland).

### siRNA transfections

Oligonucleotides for HCF-1 siRNA were purchased from Bioneer (Daejeon, Korea) and the sequences were 5′- GGCAGUGCUCUGAUUUCCAAUC -3′ (sense) and 5′- GAUUGGAAAUCAGAGCACUGCC -3′ (antisense). Scrambled control siRNA was purchased from Bioneer (SS-1011). HCF-1 siRNA duplex was dissolved in RNAse-free water and used at a final concentration of 15 pmol/well in a 24-well plate for transfection. After 24 h, cellular lysates were prepared for western blot analysis.

### Lentiviral shRNA production and infection

The lentivirus expressing the shRNA against scrambled (control) or OGT (shOGT) was produced as follows: Lentiviral pLKO.1-puro vectors encoding OGT-specific and scrambled shRNA were purchased from Sigma TRC shRNA library. The human OGT shRNA sequence (TRCN0000035064) used was 5′-CCGG-GCCCTAAGTTTGAGTCCAAAT-CTCGAG-ATTTGGACTCAAACTTAGGGC-TTTTTG-3′. The scrambled shRNA sequence (Product No. SHC002V) used was 5′-CCGG-CAACAAGATGAAGAGCACCAA-CTCGAG-TTGGTGCTCTTCATCTTGTTGTTTTT-3′. For lentivirus production, 293T cells were transfected with pLKO.1 vector along with packaging plasmids encoding Gag/Pol, Rev, and VSV-G using the Lipofectamine 2000 reagent (Invitrogen) according to the manufacturer's instructions. Culture growth media containing lentiviral particles were collected 48 h and 72 h after transfection and filtered. Viral supernatants were pooled and stored at −80°C. HeLa cells were infected with viruses in medium and selected for stable expression of shRNA by treating with puromycin (10 μg/mL) for 2 weeks.

### Cell proliferation assay

HeLa/shCTL or HeLa shOGT cells were seeded at a density of 1,000 cells/well in a 24-well plate and an 3(4,5-dimethylthiazol-2-yl)-2,5-diphenyltetrazolium bromide (MTT)-based assay was performed at the indicated times (0, 24, 48, and 72 h). HeLa cells were cultured in high- (25 mM) or low- (5 mM) glucose media. The absorbance at 570 nm was used to determine the cell proliferation rates. MTT solution (2 mg/mL) was added to each well and the plates were incubated 37°C for 2 h. The formazan crystals formed were dissolved in dimethyl sulfoxide and the absorbance of the solution was measured at 570 nm using a microplate reader (Tecan, Maennedorf, Switzerland).

### Cell invasion and colony formation assay

For invasion assay, HeLa/shCTL or HeLa/shOGT cells were seeded on Matrigel-coated Transwell inserts (Corning Incorporated, Steuben County, NY, USA) at a density of 1×10^4^ cells/well in a 24-well plate and allowed to migrate for 24 hrs. The non-migrated cells on the upper side of the Transwell filter were removed with a cotton swab, while the migrated/invaded cells in the basal side insert were fixed and stained with 4′,6-diamidino-2-phenylindole (1 μg/mL) for 10 min and rinsed three times with distilled water. Cells that migrated to the bottom side of the filter were counted in 10 fields/well under a fluorescence microscope (BX51; Olympus, Tokyo, Japan). The experiments were repeated three times. For soft agar colony formation assay, HeLa cells were infected with control or OGT shRNA, and cells (1×10^4^ cells/well) were plated in 0.3% agar media supplemented with 10% FBS, overlaid onto a previously prepared layer of 0.6% base agar. The medium was changed every 2 days. After 2 weeks, colonies were stained with 0.05% crystal violet and counted.

### Tumor xenograft

Athymic female nude mice (BALB/c-nu/nu, 5-6 weeks old; Orientbio, Seongnam, Korea; n=10) were maintained on a 12 h light/dark cycle, with food and water supplied *ad libitum*. HeLa cells treated with non-targeting (shCTL) or OGT-specific shRNAs (5 × 10^6^ HeLa/shCTL or HeLa/shOGT cells in 100 μl PBS) were injected via subcutaneous injection to the right flank of the nude mice. After the tumors became palpable, the tumor volume was measured with a caliper every week for 4 weeks. The tumor volume was calculated according to the formula: volume = (length × width^2^)/2. All animal work was performed in accordance with the Gyeongsang National University Institutional Animal Care and Use Committee (Approval No. GNU-131121-M0069).

### Immunohistochemistry analysis

Tissue samples from normal subjects, CIN2 (moderate), CIN3 (severe), or cervical cancer patients were embedded in paraffin blocks. After paraffin embedding, longitudinal sections of 5 μm were prepared. Slides were incubated with appropriate primary antibodies (Supplementary Table S2) at 4°C for 24 h. After washing three times in 0.1 M PBS, the slides were incubated with secondary antibodies (biotin-conjugated anti-rabbit or –mouse IgG) for 1 h at room temperature. Then, the slides were processed using an avidin-biotin-horseradish peroxidase (ABC) kit (Vector Laboratories, Inc. CA, USA), and finally stained with diaminobenzidine (DAB) diluted in 0.1 M PBS using a DAB substrate kit (Vector Laboratories, Inc.). Results were reviewed and verified by two observers.

### Immunofluorescence analysis

For double immunofluorescence analyses of OGT and HCF-1, we prepared placental tissues sliced in 5μm. Sectioned tissues were incubated in blocking solution (2% normal donkey serum, 0.5% Triton X-100, 0.05% sodium azide in 0.05M PBS, pH 7.4) for 1 h, followed by 4°C overnight incubation with mixed primary antibodies (anti-OGT plus anti-HCF1). After several washes in 0.1 M PBS, fluorescent labeling secondary antibodies [goat anti-Mouse IgG conjugated to Alexa Fluor 488 (Thermo, A-11029) or donkey anti-Rabbit IgG conjugated to Alexa Fluor 594 (Thermo, A-21207)] were applied, and sections were mounted with Mounting Medium including DAPI (Invitrogen, CA). Digital images were taken by fluorescence microscope (BX51-DSU; Olympus, Tokyo).

### Succinylated wheat germ agglutinin (sWGA) affinity purification

HeLa cells were lysed with RIPA lysis buffer (150mM NaCl, 50mM Tris, pH 7.4, 1mM EDTA, 0.5% Nonidet P-40) and cell lysates(~100 μg of protein) was incubated with agarose-conjugated sWGA beads (Vector Laboratories, Burlingame, CA) overnight at 4°C. For control, the inhibitory monosaccharide GlcNAc was added during sWGA-lectin-affinity purification. Precipitates were washed three times with lysis buffer and proteins were eluted by boiling in SDS sample buffer.

### Western blot analysis

Cells or tissues were homogenized in lysis buffer (50 mM Tris [pH 7.5], 150 mM NaCl, 5 mM EDTA, and 1% Nonidet P-40) and Protease Inhibitor Cocktail (Sigma-Aldrich, St Louis, MO, USA). Total proteins (10–20 μg) were resolved by SDS-PAGE and transferred onto nitrocellulose membranes (Millipore, Billerica, MA, USA). The membranes were probed with the primary antibodies listed in Supplementary Table S2. Immunoreactive antigens were detected with the Enhanced Chemiluminescence Detection Kit (Amersham Bioscience, Pittsburgh, PA, USA). The specific protein bands were analyzed by densitometry and the density values were normalized to that of the β-actin control and further analyzed using the Image J software (Bethesda, MD, USA).

### Immunoprecipitation

Protein extracts were mixed with proteinA/G agarose beads (Santa Cruz Biotechnology), incubated for 1 h at 4°C, and then centrifuged at 12,000 × *g* for 1 min. The supernatant was incubated with the immunoprecipitation (IP) antibodies overnight at 4°C and then incubated with protein A/G agarose beads for 2 h at 4°C. The negative control was prepared with protein A/G agarose beads without the antibody. The protein-bead complex was then washed and collected by centrifugation, samples were boiled in loading buffer to remove the agarose beads, and the protein (2 mg) was then separated by SDS-PAGE on 10% acrylamide gels. Proteins were then transferred to membranes, probed with antibodies against the interacting protein of interest, and processed for Western blotting as described above.

### Statistical analysis

Data are representative of three independent experiments and presented as mean ± S.E.M. Statistical analyses were performed using Student *t*-test or ANOVA. Data were considered statistically significant when *P* was less than 0.05.

## SUPPLEMENTARY TABLES


