# Supplementary material for: O-linked N-acetylglucosamine transferase promotes cervical cancer tumorigenesis through human papillomaviruses E6 and E7 oncogenes
Source: Oncotarget. 2016 Jun 16;7(28):44596–607. doi: 10.18632/oncotarget.10112 (PMC5190121; doi:10.18632/oncotarget.10112)
Supplement: Supplementary file 1 [file oncotarget-07-44596-s001.pdf]

O-linked N-acetylglucosamine transferase promotes cervical cancer tumorigenesis through human papillomaviruses E6 and E7 oncogenes

SUPPLEMENTARY TABLES

Supplementary Table S1: Baseline characteristics of the cervical cancer patients (n=30)

Variable	Value
**Age (year, median)**	57.1 (29-84)
**FIGO stage**	
IB2	2 (6.7)
IIA1	4 (13.3)
IIA2	2 (6.7)
IIB	12 (40)
IIIA	2 (6.7)
IIIB	6 (20)
IVA	0
IVB	2 (6.7)
**Histology**	
Squamous cell carcinoma	28 (93.3)
Adenocarcinoma	2 (6.7)
**SCC Ag**	30.45 (1.3-136.68)
**Hemoglobin count (g/dL)**	10.5 (8.1-13.3)
**Lymph node metastasis**	
No	10 (33.3)
Yes	16 (53.3)
Unknown	4 (13.3)

SCC-Ag: Scuamous cell carcinoma antigen
Values are presented as number (%).
FIGO, International Federation of Gynecology and Obstetrics.

Supplementary Table S2: List of primary antibodies used in this study

Antibody	Company	Catalog No.	Dilution	Applications	Source
HPV18E6(G-7)	Santa Cruz	sc-365089	1:2000, 1:500	WB,IHC	Mouse
HPV16E6/18E6	Santa Cruz	sc-460	1:1000, 1:100	WB,IHC	Mouse
HPV18E7(F-7)	Santa Cruz	sc-365035	1:2000, 1:500	WB,IHC	Mouse
HPV16E7	Santa Cruz	sc-6981	1:1000	WB	Mouse
HCF-1	Bethyl Laboratories	A301-399A	1:2000, 1:100	WB,IP,IHC,IF	Rabbit
RL2 (O-GlcNAc)	Thermo Scientific	MA1-072	1:2000, 1:100	WB,IP,IHC	Mouse
OGT(F-12)	Santa Cruz	sc-74546	1:2000, 1:500	WB,IP	Mouse
OGT(Tl-14)	Sigma	O6014	1:2000, 1:100	WB, IHC,IF	Rabbit
Anti-FLAG M2	Sigma	F1804	1:2000, 1:500	WB,IP	Mouse
β-actin	Sigma	A5441	1:10000	WB	Mouse
Ki-67	Dako	M7240	1:100	IHC	Mouse
